# Successful Sphincter-Preserving Resection of a Giant Rectal Schwannoma Using Transanal Total Mesorectal Excision: A Case Report

**DOI:** 10.70352/scrj.cr.25-0786

**Published:** 2026-03-06

**Authors:** Mamoru Miyasaka, Koichi Teramura, Yuki Okawa, Sho Sekiya, Toshihiro Kushibiki, Daisuke Saikawa, Satoshi Hayashi, Yoshinori Suzuki, Masaya Kawada, Yo Kawarada, Shuji Kitashiro, Kichizo Kaga, Shunichi Okushiba, Satoshi Hirano

**Affiliations:** 1 Department of Surgery, Tonan Hospital, Sapporo, Hokkaido, Japan; 2 Department of Gastroenterological Surgery II, Hokkaido University Faculty of Medicine, Sapporo, Hokkaido, Japan

**Keywords:** rectal schwannoma, transanal total mesorectal excision, TaTME, intersphincteric resection, sphincter preservation, benign rectal tumor

## Abstract

**INTRODUCTION:**

Rectal schwannomas are rare, accounting for less than 10% of all gastrointestinal schwannomas. When they are large and located in the lower rectum, achieving complete resection while preserving sphincter function can be technically challenging. Transanal total mesorectal excision (TaTME) provides an enhanced view of the deep pelvis and facilitates precise dissection around the anorectal junction. We report a case of a giant rectal schwannoma that was successfully resected with anal preservation using TaTME.

**CASE PRESENTATION:**

A 30-year-old woman was referred with an 11-cm rectal tumor in the pelvis identified during pregnancy. MRI revealed a well-circumscribed mass surrounding the lower rectum, in broad contact with the uterus, vagina, and anal sphincter. Endoscopic ultrasound-guided fine-needle aspiration demonstrated a benign schwannoma. Considering the patient’s strong desire for sphincter preservation, laparoscopic intersphincteric resection assisted by TaTME was performed. The operative time was 317 minutes with minimal blood loss (35 mL). The tumor was completely resected with negative margins, and no diverting stoma was required. Pathology confirmed a plexiform schwannoma without malignancy. The postoperative course was uneventful, and the patient was discharged on POD 12. At 3 months postoperatively, anal function was satisfactory, with a Low Anterior Resection Syndrome score of 14 indicating only minor symptoms.

**CONCLUSIONS:**

TaTME is a useful surgical strategy for large rectal tumors in which conventional laparoscopic approaches provide limited visualization. This approach enabled complete resection with preservation of anal sphincter function in a patient with a giant rectal schwannoma.

## Abbreviations

ESD
: endoscopic submucosal dissection
ISR
: intersphincteric resection
LARS
: Low Anterior Resection Syndrome
PDS
: polydioxanone
TaTME
: transanal total mesorectal excision

## INTRODUCTION

Schwannomas are benign peripheral nerve sheath tumors derived from Schwann cells. Within the gastrointestinal tract, they are uncommon and account for approximately 2%–6% of gastrointestinal mesenchymal tumors, occurring predominantly in the stomach.^1–3)^ Colorectal schwannomas comprise only 2%–5% of gastrointestinal schwannomas, and rectal involvement is particularly rare, with fewer than 50 cases reported in the literature to date.^4)^ Immunohistochemically, schwannomas show diffuse S-100 positivity and are typically negative for KIT and CD34, distinguishing them from gastrointestinal stromal tumors and other mesenchymal neoplasms.^2–4)^ For small rectal schwannomas, endoscopic resection techniques, including ESD, have been reported as feasible treatment options when adequate margins can be safely secured.^5,6)^ However, endoscopic approaches are generally unsuitable for large tumors, particularly those located in the lower rectum, because of the risk of incomplete resection and perforation. Large rectal schwannomas therefore usually require surgical resection. Nevertheless, conventional surgical approaches, including open, laparoscopic, and robotic surgery, may be technically challenging for tumors occupying the deep pelvis. These challenges include restricted visualization within the narrow pelvic cavity, difficulty in securing an adequate distal resection margin under direct vision, and the potential compromise of anal sphincter preservation, especially for tumors extending close to the anal canal.^4,5)^

TaTME was developed to overcome these limitations by providing a direct, magnified view of the distal rectum from a transanal perspective. This approach facilitates precise dissection in the deep pelvis and allows secure distal margin control while preserving the anal sphincter.^7–10)^ Herein, we report a case of a giant rectal schwannoma successfully resected using TaTME, achieving complete tumor removal with preservation of anal sphincter function.

## CASE PRESENTATION

**Fig. 1:**
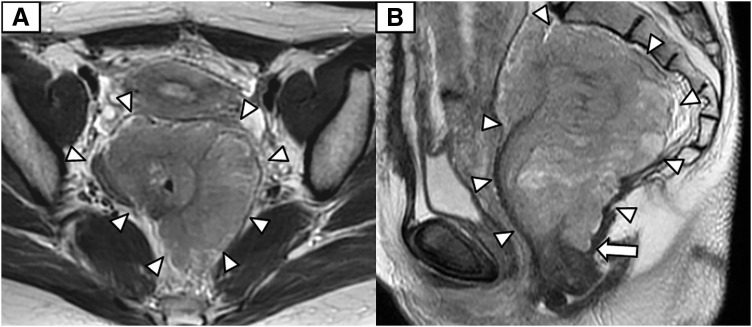
Pelvic MRI. T2-weighted images show a well-circumscribed, low-signal-intensity mass circumferentially surrounding the lower rectum. (**A**) Axial view shows the lesion in broad contact with the uterus. (**B**) Sagittal view shows the lesion extending dorsally and caudally toward the anal verge (white arrow). White arrowheads indicate the tumor margins.

**Fig. 2:**
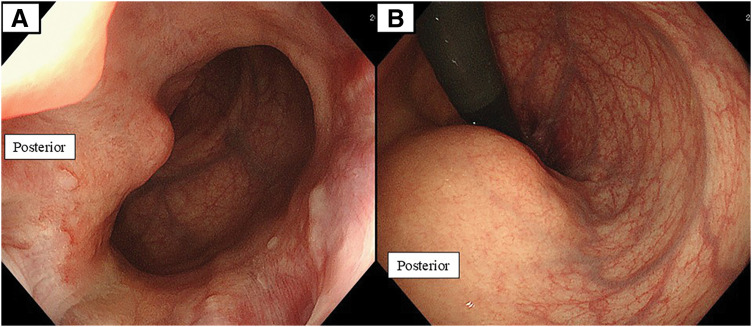
Colonoscopic findings. (**A**) Endoscopic view shows a submucosal-type bulge on the posterior rectal wall just above the anal canal. (**B**) Retroflexed view shows the same submucosal elevation on the posterior rectal wall.

A 30-year-old woman was referred for an 11-cm rectal tumor that was incidentally detected on abdominal ultrasonography at 26 weeks of gestation. MRI demonstrated a well-circumscribed mass circumferentially surrounding the lower rectum and broadly contacting the uterus, posterior vaginal wall, and anal sphincter (**Fig. 1**). Colonoscopy revealed a submucosal elevation with intact mucosa, allowing easy passage of the endoscope (**Fig. 2**). Endoscopic US-guided fine-needle aspiration revealed spindle cells consistent with a benign schwannoma. Given the patient’s strong desire to preserve anal function, laparoscopic ISR assisted by TaTME was planned. The patient delivered a healthy infant by cesarean section at 38 weeks’ gestation, and definitive surgical resection was performed 5 months after the initial diagnosis.

**Fig. 3:**
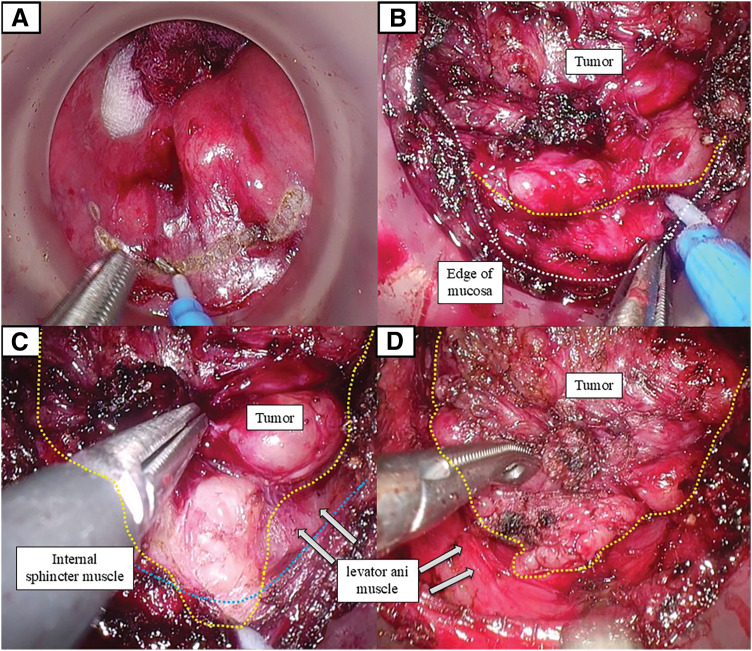
Intraoperative views during transanal total mesorectal excision. (**A**) Transanal view shows the posterior submucosal tumor and the distal mucosal incision line established under direct endoscopic visualization. (**B**) Posterior transanal dissection after circumferential mucosal incision. The incised distal mucosal edge and internal anal sphincter were retracted caudally, exposing the tumor within the intersphincteric space toward the anal verge. Yellow dotted lines indicate the tumor margin, and white dotted lines indicate the mucosal incision line. (**C**) Further posterior transanal dissection after exposure of the tumor margin. The dissection plane between the tumor and the levator ani muscle is clearly visualized. The tumor margin is outlined by yellow dotted lines, and the internal anal sphincter muscle by the blue dotted line. (**D**) Continuation of transanal posterior dissection. The relationship between the tumor and the levator ani muscle is further delineated as dissection proceeds cranially along the tumor surface.

Surgery was performed using a coordinated 2-team approach. During the transanal phase, the tumor was identified as a submucosal lesion on the posterior rectal wall just above the anal canal. Circumferential mucosal incision was initiated approximately 3 cm from the anal verge, which was determined intraoperatively based on direct perineal measurement and digital palpation. Following closure of the rectal lumen with a purse-string suture, circumferential dissection of the rectal wall was performed. On the posterior wall adjacent to the tumor, the lesion was immediately exposed upon incision of the internal anal sphincter. The tumor extended dorsally between the internal and external anal sphincters. Gentle caudal traction of the incised distal mucosa and internal anal sphincter allowed clear exposure of the tumor margin, facilitating controlled dissection along the tumor surface while preserving the internal anal sphincter (**Fig. 3**). Dissection was then continued cephalad along the tumor margin within the limited pelvic space.

**Fig. 4:**
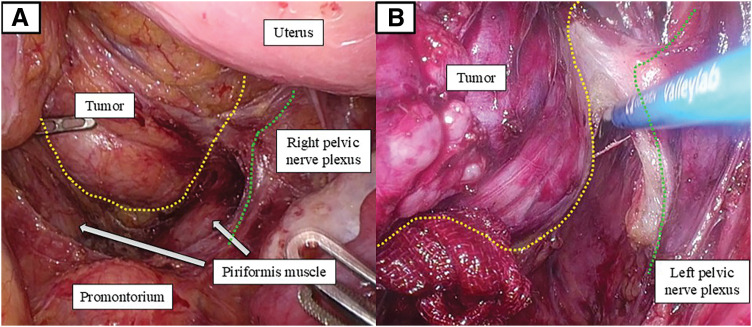
Intraoperative views during the abdominal and transanal phases. (**A**) Transabdominal view during the abdominal phase. Pelvic dissection advanced to approximately the S3 level, slightly caudal to the piriformis muscle. The tumor margin is outlined by a yellow dotted line, and the pelvic nerve plexus by a green dotted line. Although the peritoneal reflection could be identified, further distal dissection was limited by the bulky tumor occupying the deep pelvis, resulting in restricted visualization from the abdominal approach. (**B**) Transanal view during cephalad dissection along the tumor. Dissection continued cranially along the tumor surface (yellow dotted line) while carefully maintaining the boundary with the pelvic nerve plexus (green dotted line). The dissection plane created during the abdominal phase was confirmed intraoperatively, allowing safe mobilization of the tumor while preserving the pelvic autonomic nerves.

During the abdominal phase, the splenic flexure of the left colon was mobilized, and the inferior mesenteric artery was divided near its origin to facilitate tension-free reconstruction, without oncologic lymphadenectomy. The mesorectum was dissected along the proper avascular plane as far distally as feasible. Although the peritoneal reflection on the anterior wall could be identified, further distal pelvic dissection and precise visualization of the distal rectum were limited by the bulky tumor occupying the deep pelvis. The extent of pelvic dissection achievable from the abdominal approach and its relationship to the pelvic autonomic nerves is shown in **Fig. 4A**. Subsequent cephalad dissection along the tumor margin under direct transanal visualization enabled confirmation of the abdominal dissection plane and safe preservation of the pelvic nerve plexus, allowing the 2 approaches to rendezvous in the appropriate mesorectal plane (**Fig. 4B**), thereby completing circumferential mobilization of the rectum.

The specimen was delivered through a small umbilical incision. After extracorporeal division of the proximal rectum with adequate margins, the remaining sigmoid colon was anastomosed to the anal canal using a single-stapling technique with a 29-mm circular stapler. The anastomotic ring was complete and airtight, and additional reinforcing sutures were placed per anus under direct vision using interrupted 3-0 PDS sutures (16 stitches). Operative time was 317 min, with an estimated blood loss of 35 mL; no diverting stoma was required.

**Fig. 5:**
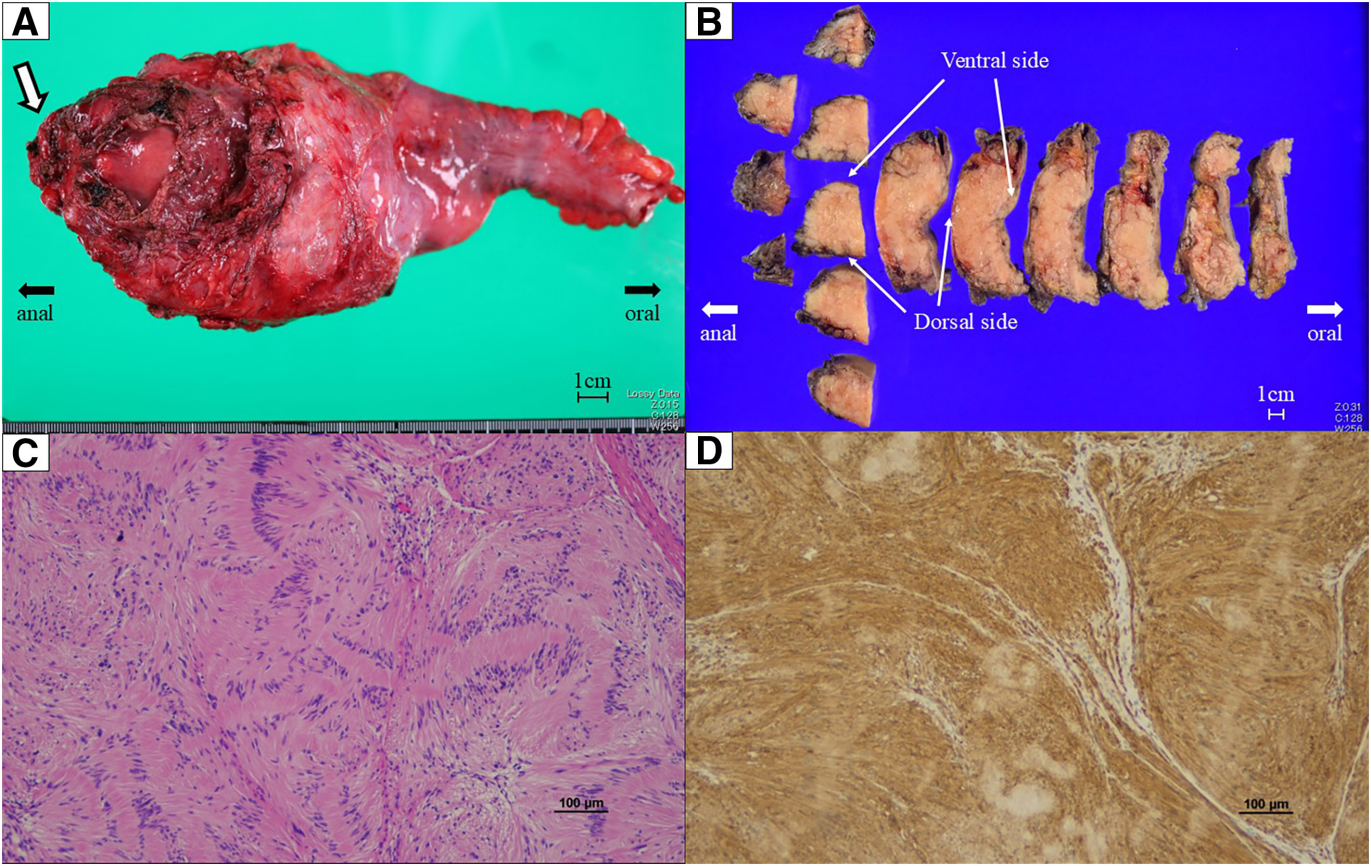
Gross pathological findings. (**A**) Fresh specimen shows a large rectal submucosal mass in the lower rectum extending caudally toward the anal verge. The white arrow indicates the portion protruding dorsally and caudally. (**B**) Formalin-fixed specimen was serially sectioned to demonstrate the relationship between the tumor and the rectal wall. Longitudinal sections along the rectal axis were obtained on the anal side, whereas cross-sectional slices were created on the oral side to illustrate the extent of the lesion and the resection margins. The ventral and dorsal aspects are indicated. (**C**) Hematoxylin and eosin staining showing a benign spindle-cell tumor with a multinodular growth pattern. (**D**) Immunohistochemical staining demonstrating diffuse S-100 positivity in tumor cells.

Gross examination revealed a multinodular, encapsulated mass measuring 130 × 90 × 50 mm. Histologically, the tumor was diagnosed as a plexiform schwannoma, showing diffuse S-100 protein positivity on immunohistochemical staining (**Fig. 5**). All resection margins were microscopically free of tumor; however, precise evaluation of the distal margin was partially limited by cauterization of the anal mucosal edge. The proximal resection margin measured 10 cm. The postoperative course was uneventful, and the patient was discharged on POD 12. At 3 months after surgery, anal function was satisfactory without incontinence, with a LARS score of 14.^11–13)^ Postoperative urinary function was preserved, with no urinary retention or voiding difficulty reported during hospitalization or at follow-up.

## DISCUSSION

Rectal schwannomas are extremely rare, accounting for only a small fraction of gastrointestinal schwannomas, and their management poses unique surgical challenges. Complete resection with negative margins is recommended because local recurrence has been reported even in benign lesions.^1–3)^ Although the tumor in the present case was detected during pregnancy, there is no evidence suggesting that pregnancy-related hormonal changes influence the growth of gastrointestinal schwannomas. However, when these tumors are located in the lower rectum, achieving adequate margins while preserving sphincter function is technically demanding.^4,5)^ In the present case, the tumor extended close to the anal canal and projected dorsally and caudally, a configuration that would have made secure distal margin control difficult using a conventional transabdominal approach alone.^6)^ The TaTME approach was advantageous because it provided a direct, magnified view of the distal rectum, allowing precise dissection along the tumor surface while preserving the anal sphincter.^7–9)^ Previous reports by Lim et al. and Hasegawa et al. have also demonstrated the usefulness of transanal approaches for benign or large rectal lesions requiring deep pelvic access.^10,14)^

Histologically, the tumor was diagnosed as a benign schwannoma of the plexiform type, without any malignant features, confirming the appropriateness of a sphincter-preserving strategy.^15)^ Plexiform schwannoma is a rare histologic variant characterized by a multinodular growth pattern and is generally considered benign. Compared with conventional schwannomas, its complex architecture may occasionally make complete excision technically challenging. Although malignant transformation is exceedingly rare, local recurrence has been reported after incomplete resection, underscoring the importance of en bloc removal with negative margins. Given the benign nature of plexiform schwannoma and the absence of malignant features, intensive postoperative surveillance was not considered mandatory. However, periodic clinical assessment may be reasonable, particularly in cases with limited surgical margins.

Given the benign nature of the lesion and the patient’s strong wish to avoid a permanent stoma, ISR assisted by TaTME was selected to achieve complete excision with negative margins while maximizing functional preservation. Wide lateral dissection was unnecessary, allowing preservation of the hypogastric and pelvic splanchnic nerves, as demonstrated by the correspondence between the abdominal and transanal dissection planes (**Fig. 4**).

Intraoperatively, posterior dissection was particularly challenging because the tumor extended beyond the initially planned distal mucosal incision toward the anal verge. Gentle caudal traction of the incised distal mucosa and internal anal sphincter enabled clear exposure of the tumor margin and the plane between the tumor and the levator ani muscle, facilitating controlled dissection along the tumor capsule while preserving sphincter structures (**Fig. 3**). This transanal view allowed safe en bloc resection with negative margins without rectal perforation or sphincter injury.

Postoperatively, the patient’s bowel function was well preserved, with a LARS score of 14, indicating only minor symptoms.^11–13)^ This favorable outcome suggests that TaTME-assisted ISR can achieve both complete excision and satisfactory functional results even for large benign rectal tumors in the deep pelvis. Nonetheless, the procedure should be performed by surgeons experienced in both TaTME and pelvic dissection techniques due to its technical complexity and potential risk of pelvic nerve injury.

## CONCLUSIONS

In summary, TaTME-assisted ISR enabled complete removal of a giant rectal schwannoma while preserving anal sphincter function. This technique represents a valuable option for large benign tumors of the lower rectum when conventional laparoscopic approaches provide limited visualization. Careful case selection and technical expertise are essential to ensure complete tumor resection and optimal functional outcomes.

## DECLARATIONS

### Availability of data and materials

All data generated or analyzed during this study are included in this article. Additional information is available from the corresponding author upon reasonable request.
